# Supplementary material for: Are carbon emissions trading and green financial instruments synergistic? -Comprehensive quantitative research based on content analysis
Source: PLoS One. 2024 Mar 7;19(3):e0298601. doi: 10.1371/journal.pone.0298601 (PMC10919601; doi:10.1371/journal.pone.0298601)
Supplement: S1 Data — (ZIP) [file pone.0298601.s001.zip › Data/地级市Z_F工作报告.zip!/╣π╢1⁄2/╜╥╤⌠╩╨/└┤╫╘╣1⁄2╓┌║┼í╛┬φ┐╦╩2╛▌═°í┐_2008.docx]

各位代表：

　　我代表市人民政府向大会作政府工作报告，请予审议，并请政协各位委员和其他列席人员提出意见。

　　2007年工作回顾

　　2007年，在省委、省政府和市委的正确领导下，在市人大及其常委会和市政协的监督支持下，市政府坚持以邓小平理论和“三个代表”重要思想为指导，全面落实科学发展观，按照党的十七大、省第十次党代会和市第四次党代会精神，狠抓发展第一要务，采取有效措施，强力组织推进，经济社会保持良性快速发展势头。全年生产总值592.27亿元，比上年（下同）增长18.1%；全社会固定资产投资201.29亿元，增长32.4%；地方财政一般预算收入17.32亿元，增长25.9%，主要经济指标完成或超额完成市四届人大一次会议确定的目标任务。

　　——经济持续较快发展

　　工业生产增长较快。规模以上工业增加值145.82亿元，增长40.7%。新增国家和省级高新技术企业8家、省级民营科技企业12家，新增驰名商标3件、著名商标17件、省名牌产品2个。大南海国际石化综合工业园获省批准设立，珠海金湾（揭阳）产业转移园区、揭阳大南山工业区等前期工作正在抓紧推进。农村经济稳步发展。第一产业增加值83.75亿元，增长4.8%。粤东柑桔良种无病毒苗木繁育中心等项目建设进展顺利；普宁蕉柑、揭东竹笋、揭西青榄等农业标准化示范区建设稳步推进。21家省、市级农业龙头企业辐射带动作用不断增强，农产品批发市场逐步配套。玉湖经验得到大力推广和落实，新农村建设步伐加快。内外经贸有效拓展。社会消费品零售总额208.92亿元，增长23.3%；外贸出口总额17.69亿美元，增长25%；实际利用外资1.53亿美元，增长31.2%。成功举办第六届玉器节、第二届中药材交易会、揭西生态旅游文化节和揭阳轻工产品（郑州）展销会、五金不锈钢制品博览会等节会，隆重举行纪念建市15周年暨招商引资大会，签约和开工奠基剪彩项目203个，投资总额368.28亿元。闽粤赣十三市和粤东四市经济社会协作深入开展，与泰国南邦市、韩国务安郡等地交流合作进一步加强。

　　——投资建设力度加大

　　重大项目取得突破。惠来电厂1、2号机组及7万吨级码头建成投产，3、4号机组正在开展前期工作；揭阳潮汕机场获国家批准建设，征地拆迁等工作正在抓紧进行；厦深铁路已开工建设。LNG（液化天然气）项目已着手建设2个卫星站；大型核电项目初可研工作基本完成。亚洲资本论坛全球会议中心、中国（揭阳）阳美玉都广场已动工；大中华韩国城、热电联产、大型造纸等项目正在抓紧落实。基础设施建设加快。国道206线曲丰段、省道236线池揭段、省道335线霖磐至浦东段等大修工程建设顺利实施；全市81%行政村实现通水泥路目标。220千伏紫峰、铁山输变电工程建成投产，500千伏榕江等输变电工程建设步伐加快。三洲拦河闸工程已动工建设，榕城围、渔湖围等防洪工程加快推进。城区建设成效明显。市区30亿工程正在加紧实施，北河大桥整治工程顺利完成，揭阳学宫广场、西凤大桥新建、南河大桥扩建、淡浦南路和市区污水处理厂等项目建设步伐加快；各县（市、区）筹资1亿元以上投入城区市政公共设施建设任务得到落实，市政功能逐步配套。

　　——各项事业协调推进

　　教育事业发展加快。筹措教育资金5.97亿元，新改扩建中小学81所126.91万平方米，新招聘教师3059名；成功与北师大合作设立教师教研中心揭阳教育研究院，专门组织55名高中校长、213名初中校长集中培训，组织9656名学历未达标教师接受大专以上学历教育；高考上省线学生1.78万人，其中上本科线7804人，增长25.1%。揭阳学院顺利通过高职高专院校人才培养工作水平评估。卫生工作取得成效。疾病预防控制和妇幼保健工作扎实推进，公共卫生应急处理能力有所提高。打击非法组织献血活动成效明显，无偿献血工作得到加强。新型农村合作医疗制度全面实施，参加人数328.6万人，覆盖率74.3%。“一村一站一医生”建设扎实开展，1445个行政村卫生站全面建成。其他事业协调推进。“八荣八耻”社会主义荣辱观和 “爱国、守法、诚信、知礼”现代公民道德教育活动深入开展，揭阳学宫修缮工程全面启动，揭阳先贤揭猛研究会正式成立。“百龙闹榕江，万众迎奥运”龙舟竞赛活动成功举办，农村体育场地建设步伐加快。市级电子政务平台和企业基础信息共享首期工程顺利建成。审计监督得到加强。物价、统计、旅游、气象、地震、人民防空、国防动员、民族宗教、对台事务、文史档案、广播电视、新闻出版等事业取得新进步。

——社会保持和谐稳定

　　生态管理力度加大。人口与计生管理不断加强，完成省下达的目标任务。环保执法力度加大，市电镀工业区和30家严重污染企业被依法关停。污染源普查前期工作顺利完成，开展“榕江环保一路行”活动，公众环境意识不断增强。乱开乱采资源行为受到严厉打击，森林、矿产、土地等资源管理得到加强。市场秩序不断规范。产品质量和食品、药品安全专项整治和打假工作取得成效。“黑网吧”现象得到有力打击。交通运输、危险化学品、消防安全等专项整治深入开展，安全生产形势保持稳定。治安状况逐步好转。严打整治斗争深入开展，重点地区和突出问题得到集中整治，被省评为“社会治安综合治理工作优秀市”。

　　——人民生活不断改善

　　统筹就业有效推进。参加百万农村青年技能培训和农民工技能提升培训2.1万人、退役士兵就业培训1097人，新增就业2.1万人，农村劳动力转移就业6.3万人，城镇登记失业率控制在3%以下。城镇居民人均可支配收入10751元，增长9%；农村居民人均纯收入4561元，增长5.2%。社会保障不断完善。企业养老保险费收入3.05亿元，增长10.4%。企业离退休人员基本养老待遇得到较大提高，人月均增加115元。基本医疗保险工作进展顺利，城镇居民参保人数28.32万人；城镇职工参保人数11.7万人，增长30%。城乡居民最低生活保障水平逐步提高，人月均补助标准分别提高到80元以上、40元以上。扶贫助困扎实开展。扶贫开发力度加大，投入扶贫资金8000多万元，有效地促进了山区、贫困地区发展。“献爱心、送温暖”捐助活动深入开展，捐赠款物600多万元，救助困难群众3.3万人次。赈灾救济工作得到加强，社会救助网络逐步完善。

　　——自身建设得到加强

　　民主建设进程加快。定期向人大及其常委会报告工作、向政协通报情况的制度进一步完善，人大及其常委会的决议、决定得到认真执行。与工、青、妇等群团组织的联系更加紧密，社会各界的意见建议得到重视和吸纳，政府决策的科学化、民主化水平不断提高。全年办理市人大代表议案、建议46件，政协委员提案、建议意见138件。网上“市长信箱”受理群众来信4583件，回复率100%。村（居）民自治制度逐步完善，政务、村务、厂务公开顺利实施。法制建设有效推进。行政执法责任制得到落实，法制监督力度加大，行政机关执法行为进一步规范。行政复议工作得到加强，行政纠纷得到有效化解。“五五”普法宣传深入开展，全民法律意识不断增强。政风建设得到加强。市政府五项廉政承诺得到有效履行，廉政建设责任制进一步落实。全市行政审批电子监察系统顺利开通，行政审批透明度进一步提高。群众来信来访及时妥善处置，人民群众反映强烈的热难点问题得到有效解决。商业贿赂等专项整治扎实开展，部门和行业不正之风逐步得到纠正。

　　各位代表，过去一年，我市经济社会发展取得了显著的成绩。这是省委省政府和市委正确领导的结果，是各级人大及其常委会和政协监督支持的结果，是全市人民团结拼搏、共同努力的结果，是中央和省驻揭单位、驻揭人民解放军、武警官兵、人民警察、各民主党派、人民团体、港澳台同胞、海外侨胞和友好人士关心支持的结果。在此，我谨代表市人民政府表示衷心的感谢并致以崇高的敬意！

　　在肯定成绩的同时，我们也清醒地看到，我市经济社会发展仍存在不少困难和问题。突出表现在：经济总量仍然不大，人均水平差距明显；产业结构不够合理，整体经济实力不强；企业规模普遍偏小，自主创新能力较弱；基础设施有待继续完善，投资环境仍不够理想；城镇建设步伐缓慢，县域经济发展滞后；各项事业欠账较多，社会文明程度有待提高；市场秩序仍需进一步整顿和规范，治污保洁和节能减排任务繁重；社会治安形势依然严峻，维稳压力较大；公务员队伍思想还不够解放，行政效能尚需进一步提高。对此，我们将采取更加有效措施，在加快发展中认真解决。

2008年工作安排

　　2008年是实现“三年打基础、五年大变化、十年大发展”奋斗目标的关键一年。市政府工作的指导思想是：高举中国特色社会主义伟大旗帜，以邓小平理论和“三个代表”重要思想为指导，深入贯彻科学发展观，全面落实党的十七大、省委十届二次全会、市委四届四次全会精神，按照“解放思想，创新机制，完善规划，增强信心，抢抓机遇，艰苦奋斗，科学发展，十年赶上全省发展平均水平”的总体要求，坚持解放思想和加快发展，坚持改革开放和自主创新，坚持调整结构和提高质量，坚持节约资源和保护环境，坚持改善民生和促进和谐，着力推进经济建设、政治建设、文化建设、社会建设，促进经济社会又好又快发展。

　　今年我市经济社会发展的主要预期目标：生产总值增长19%，单位生产总值能耗降低3%，二氧化硫和化学需氧量分别削减12.4%和3%，社会消费品零售总额增长25%，全社会固定资产投资增长32%，外贸出口增长18%，地方财政一般预算收入增长22.7%，居民消费价格指数控制在3%左右，城镇登记失业率控制在3.4%以内，城镇居民人均可支配收入增长9%，农民人均纯收入增长6%，人口自然增长率控制在7.4‰以下。

　　实现上述目标，必须突出抓好十方面工作：

　　一、抓好自主创新，推动产业升级

　　强化自主创新。认真落实鼓励自主创新的政策措施，切实搞好科技服务和创新平台建设。大力培植高新技术企业和民营科技企业，支持设立省级以上企业工程技术研发中心，加强产学研结合，加速科技成果转化。力争新增高新技术企业6家、省级民营科技企业12家、市级以上工程技术研发中心2个。

　　推进产业集群。推进省市共建支柱产业基地建设，做大做强五金机械、化工塑料、纺织服装、食品医药等传统产业，大力引进和发展电力能源、石油化工、装备制造等重化工业和电子信息等高新技术产业，加快现代产业体系建设，确保全年工业增加值增长23.3%。认真筹建普宁服装研发培训中心，抓紧完善服装、珠宝玉器省级检验站，争取尽快设立不锈钢制品国家检验中心，为产业集群发展提供技术支撑。

　　壮大工业规模。加大对中小企业的扶持和引导，落实具体扶持措施，鼓励企业增资扩产，力促规模下企业升级转为规模上企业，培植更多规模以上工业企业，确保新增投资500万元以上工业项目250个，净增规模以上工业企业360家。

　　发展龙头企业。进一步完善领导挂钩重点企业制度，着力打造12条“经济大船”。大力扶持榕泰化工城、康美中药物流港、巨轮高精度液压硫化机等重点项目建设，壮大龙头企业经营实力。积极引导企业实行股份制改造，推动优质企业加快上市步伐，力争新增3家以上上市公司。

　　打造品牌产品。引导和支持创建区域品牌，积极鼓励企业申报品牌和商标，争创一批品牌产品，力争新增驰名商标2件、国家免检产品2个、名牌产品4个、著名商标12件。

　　加快园区建设。大力推进大南海国际石化综合工业园、珠海金湾（揭阳）产业转移园区、惠州陈江（揭阳）产业转移园区、空港经济区、大南山工业区等工业基地规划建设，认真搞好各县（市、区）工业园区建设和管理，为工业大发展夯实载体。

　　二、抓好重点项目，完善基础设施

　　加快交通建设。着力搞好揭阳潮汕机场建设前期工作，7月底前完成征地拆迁安置等项目建设，确保按期动工。加快国道206线曲丰段和曲溪至小坑段、省道335线深坑至曲溪段、省道236线池揭段大修和南河大桥扩建工程建设，抓紧开展国道324线普宁石桥头至东埔平交段、惠来沿海一级公路、河婆至五云段等项目建设前期工作。积极推进汕揭、潮揭、汕头—普宁—揭西、揭阳至惠州高速公路揭阳段建设，抓紧做好揭东至惠来高速公路揭阳段、神泉港10—30万吨级码头规划等工作。积极配合搞好厦深铁路建设，加快县通镇、镇通行政村公路建设改造，确保按期完成任务。

加快能源建设。大力推进惠来电厂建设，争取3、4号机组尽快获准动工。积极争取LNG接收站、大型核电等重大项目落户我市。加快揭东开发区、玉滘2个LNG卫星站建设，尽快启动城市天然气利用工程。抓紧推进500千伏榕江输变电、惠来电厂送电线路等工程建设，确保按期建成投产。继续推进农村电网改造与建设，改善电网结构，满足城乡用电需求。

　　加快水利建设。抓好榕江大围项目打捆上报跟踪落实，加快三洲拦河闸应急重建工程建设，推进揭东磐岭围、普宁流沙联围新河堤、揭西象山拦河闸、惠来赤岑水闸和揭东、惠来县城防洪工程等项目建设，认真实施列入省人大议案的小型水库除险加固、电排站改造等工程，提高城乡水利防灾减灾能力。

　　三、抓好城区建设，打造核心区域

　　加强规划控制。继续抓好城镇体系规划编制工作，完善市区总体规划和规划建成区控制性详细规划，抓紧完善50平方公里新城区总体规划。加快百里中华历史文化景观长廊规划设计与实施。加强市区、县（市）城区土地控制和规划开发，促进城区规范有序发展。

　　加快市政建设。抓紧推进市区30亿工程，争取莲花大桥立项建设，加快榕城围南北堤拆迁和淡浦南路、西凤大桥等市政项目建设步伐，启动玉浦、凤潮片区市政工程建设。认真搞好旧城开发和城中村改造。积极引导市区房地产开发，着力改善人居环境。继续督促各县（市、区）落实筹资1亿元以上投入城区市政公共设施建设任务，努力改变城区面貌。

　　搞好经营管理。深化投融资体制改革，采用BOT、TOT等形式，多渠道筹措建设资金。盘活政府公共资源，提高公共投资效益。尽快理顺市区管理体制，深化市政公用事业改革。加大城镇管理执法力度，深入开展交通、环卫等专项整治，坚决取缔市区机动三轮车，刹住和清理拆除违章建筑，大力整治“脏、乱、差”现象，树立良好城市形象。

　　四、抓好市场开拓，加强对外合作

　　搞旺商贸流通。大力发展现代服务业，确保新增87家限额以上商业企业。积极组织企业参加各类经贸洽谈活动，继续办好玉器、衬衣暨纺织服装、五金不锈钢制品等节会，扩大区域品牌效应。认真实施城市商业网点规划，加快中国（揭阳）阳美玉都广场、宝德电脑数码广场、东山金城商业文化步行街和普宁高乐玩具城、粤东富民国际服装城等建设，配套完善揭阳卷烟物流配送中心、五金不锈钢制品交易中心、普宁粤东卷烟物流配送中心等流通载体，扩大消费需求。

　　大力招商引资。实行招商引资一把手工程，落实以县为主的招商责任，强化招商责任考核，争取在引进重大项目上有更大的突破。力促省物资集团粤东物流中心、大中华韩国城、热电联产、造纸等重大项目尽快落户我市。充分发挥市政府经济顾问的作用，重点瞄准大企业、大财团开展招商，引进更多更好的项目、资金和技术。积极实施“乡贤回归”工程，大力吸引在外揭籍企业家回乡投资创业。狠抓项目跟踪落实，推动招商项目尤其是2005年以来签约及奠基开工1亿元以上重点项目顺利落户建设。确保全年实际利用外资增长33.6%。

　　发展对外经贸。应对出口退税政策调整，优化出口产品结构，提高外贸出口效益。大力鼓励企业开展国际化经营，重点扶持纺织服装、五金不锈钢等优势企业设立境外销售网络。积极开拓东南亚、拉美、非洲等市场，拓宽国际贸易渠道。加强与海关、检验检疫、外汇管理、税务等部门的协调沟通，加快电子口岸建设，做好一类口岸申报跟踪落实，优化“大通关”环境。

　　加强经贸协作。进一步加强与泰国南邦市、韩国务安郡等地交流与合作，争取启动具体合作项目。积极参与海峡西岸经济区、闽粤赣十三市、粤东四市经济社会协作，促进区域共同发展。

　　五、抓好农业生产，繁荣农村经济

　　发展现代农业。严格保护耕地，稳定提高粮食生产。加强农田水利设施建设，解决农业生产灌溉问题。加快现代农业示范园区建设，大力发展特色、效益和生态农业，提高农业综合效益。扶持农业龙头企业发展，推进农副产品深加工，配套完善农产品批发市场，健全农业生产服务体系。鼓励创立农业品牌，推广标准化生产，加强农产品质量安全监测。加快现代畜牧业、现代渔业和现代林业发展。

推进新农村建设。认真办好71个新农村建设示范点，加快农村基础设施建设和旧村改造，改善农村生产生活条件。强化农村环境综合整治，促进农村净化、美化，积极创建生态和谐文明村，加快社会主义新农村建设进程。

　　推广玉湖经验。大力推广和落实玉湖经验，加强农村基层政权建设。落实农村干部退休退职基金制度，逐步改善基层干部待遇。稳定农村土地承包制度，引导土地承包经营权依法有序流转。完善村账镇管和审计监督制度，加强农村集体资产和财务管理，探索化解镇村集体债务的有效办法。

　　培育新型农民。加强农民知识技能培训，提高农民就业创业能力，扩大农村富余劳动力转移就业规模。加大乡土人才、民间艺人的开发和评价力度，大力培育新型农民，发挥农民建设新农村的主体作用。

　　六、抓好财税社保，增强财力保障

　　强力组织征缴。认真抓好税源调查，深入挖掘增收潜力，培育壮大地方税源。充分发挥企业基础信息共享工程的作用，加大重点税种征收力度，促进税费收入持续增长。规范非税收入征管，逐步提高税收收入占地方财政收入、地方财政收入占GDP的比重。完善企业社保费征缴机制，强化社保扩面征缴，确保全年企业养老保险费增长15%。

　　严格支出管理。调整优化财政支出结构，集中财力保障“三农”、文教卫生、社会保障等领域资金支出需要。继续深化财政管理体制改革，全面落实部门预算、国库集中支付等制度，加快建立公共财政体系框架。完善社保基金后续管理，做实个人账户，严格基金支出程序，确保基本养老金按时足额发放。

　　加强检查监督。强化稽查手段，严厉打击偷税、逃税等行为，坚决堵塞税收漏洞，确保依法征收、应收尽收。加强财政预算执行监督，深入开展财政绩效评价工作，强化审计监督，确保资金安全、专款专用。

　　做好金融工作。加强银企合作，加大金融机构对地方发展的支持力度，争取中小企业贷款担保公司尽快获准开展业务。积极稳妥推进农信社试点改革，做好停业整顿城信社退市工作，加大金融资产追收处置力度，促进地方金融产业规范高效发展。

　　七、抓好各项事业，推动社会进步

　　优先发展教育。继续落实教育资金筹措、校舍建设和提高师生素质三大任务。认真落实城乡免费义务教育政策，抓好防流控辍工作，巩固提高普及义务教育成果。加快学校基础设施建设，完成761所规范化学校创建任务。大力发展职业技术教育，促进和规范民办教育。继续鼓励在职教师报考北师大教育硕士研究生，加强中小学校长培训和教师学历达标教育，建立健全校长绩效考评机制，大力招聘引进高学历优秀教师。设立扶持贫困家庭大学生基金，逐步解决贫困家庭子女读大学难问题。加强教师职业道德和学生素质教育，提高师生思想道德水平。

　　加快文化建设。加强公民道德教育，提升公民道德水平。深入开展精神文明创建活动，积极创建文明城市、文明村（居）。做好揭阳学宫修缮工作，搞好文物、文化遗产的保护和开发。大力发展特色文化产业和文化事业。加强文化市场管理。抓紧建设市文化中心，加快揭阳学宫广场、东湖公园等建设步伐。加强基层文化建设，发展公益性文化事业，丰富城乡群众文化生活。

　　加强卫生工作。着力推进中医药大市建设，加快市中医院等医疗单位“中医名院”创建步伐，积极开展农村中医工作先进县（市）创建活动。加强卫生监督，强化疾病预防控制。做好无偿献血工作，保障临床用血需要。抓好市人民医院、红十字会慈云医院住院大楼和市卫校新校区等建设，继续健全城乡医疗卫生服务体系。完善新型农村合作医疗制度，确保全市覆盖率达92%。

　　促进其它事业。深入开展“全民健身与奥运同行”活动，大力发展群众体育和竞技体育。加强国防教育，做好双拥工作。落实民族宗教政策，发挥宗教在促进社会和谐方面的积极作用。发展老龄、妇幼和残疾人事业。进一步做好外事侨务、对台、人防、史志、档案、气象、地震等工作。

　八、抓好民生民计，促进成果共享

　　努力扩大就业。认真研究制订优惠政策措施，积极鼓励本地企业招用本地劳动力。加快市技校、惠来县技校等培训基地建设，加强劳动力培训和转移输出，增加就业岗位，争取新增就业2.2万人。加强人力资源市场建设，健全公共就业服务体系和就业援助制度，重点帮助下岗失业人员、零就业家庭、农村贫困家庭、被征地农民等人员就业。规范劳动合同管理，构建和谐劳动关系。

　　健全保障体系。落实城乡居民最低生活保障制度，做到应保尽保。积极实施被征地农民养老保障办法，认真制订并落实解决早期离开国有、集体企业人员社会保险问题的政策措施，逐步建立健全社会保障体系，推进城镇职工、居民基本医疗保险工作，力争分别达12万人、28.32万人。

　　加强扶贫救济。健全社会救助体系，发展社会福利事业，完善五保供养机制。重新成立市慈善总会，充分发挥市红十字会的作用。大力实施饮水、安居等民心工程，建设经济适用房、廉租房。着力解决人民群众关心的热点、难点问题，依法保障公民的权利和利益，促进社会公平正义。

　　九、抓好各项管理，构建和谐揭阳

　　强化节能减排。落实节能减排工作责任制，加大执法监察力度。抓紧建立健全能源统计制度，加强信息采集能力建设，提高能源预测预警能力。坚持资源节约集约开发和综合利用，鼓励发展循环经济。推进建筑、商业、交通等节能，着力提高全社会节能水平。严格限制新上高耗能、高耗水、高污染项目，加快淘汰落后生产能力。大力推行清洁生产，积极发展环保产业。认真开展污染源普查，完善环保总体规划。着力搞好榕江、龙江、练江等流域水污染综合整治，深入开展“榕江环保一路行”活动，切实保护饮用水资源。抓紧编制生活污水处理厂建设专项规划，加快县（市）、镇污水处理厂、垃圾处理场等环保设施建设，提高城乡治污保洁水平。

　　强化生态保护。严格控制人口增长，稳定低生育水平，提高出生人口素质。完善殡葬设施，巩固殡改成果。严格落实土地管理和耕地保护责任，加大国土资源执法监察力度。做好第二次土地调查和新一轮土地利用总体规划修编工作，优化土地布局和结构，保障重点项目建设和城市发展需要。加大资源保护力度，严厉打击乱开乱采资源行为。积极开展创建林业生态县活动，建设绿色生态揭阳。

　　强化市场监管。落实市场价格临时监管措施，确保市场供应和物价基本稳定。严厉打击各类经济违法犯罪活动，整顿和规范市场秩序。深入开展产品质量和食品药品安全专项整治，完善食品药品安全监管长效机制。加强交通运输、危险化学品等专项整治，坚决遏制重特大安全生产事故的发生。加快社会信用建设，努力打造“诚信揭阳”。

　　强化社会管理。继续完善信访工作机制，加强人民调解工作，健全四级排查调处网络，及时调处化解基层矛盾纠纷。加强突发公共事件和防灾减灾应急管理，提高保障公共安全和处置突发公共事件的能力。全面落实防控综合治理措施，大力推广和运用治安视频监控等手段，建立健全群防群治网络。加强公安队伍建设，实行警力下沉。深入开展严打整治斗争，着力解决治安突出问题，增强群众安全感。

　　十、抓好自身建设，提高行政效能

　　坚持解放思想。转变发展观念，创新工作方式，树立世界眼光和开放观念，用科学的战略思维思考和谋划经济社会发展。更加注重改革创新，坚决冲破妨碍科学发展的思想观念，革除束缚科学发展的体制弊端，力促在关键领域改革攻坚取得新突破，以新一轮思想大解放推动大发展。

　　加快民主进程。坚持向人大及其常委会报告工作和向人民政协通报情况的制度，自觉接受监督。及时办理人大代表议案、建议和政协委员提案，认真听取民主党派、工商联、无党派人士和人民团体的意见建议。发挥市政府经济顾问、专家咨询组等作用，健全行政决策机制，推进政府决策科学化、民主化。完善政府新闻发言人制度，大力推进政务公开，增强政府工作的透明度。

推进依法行政。贯彻国务院《全面推进依法行政实施纲要》，提高依法行政水平。规范行政执法，加强执法监督。创新行政复议方式，加强行政复议工作。继续深化行政管理体制改革，精简和规范行政审批。认真开展普法教育，增强全民法制观念。

　　加强机关建设。大兴求真务实之风，深入调查研究，精简会议文件，狠抓决策部署的检查落实。加快电子政务建设，提高行政效能。深入开展廉政建设和反腐败斗争，落实廉政建设责任制，坚决纠正损害人民群众利益的不正之风。加强审计和监察工作，严肃查处违法违纪案件，纠正各种违规行为。加强公务员教育培训，提高队伍整体素质，努力建设服务政府、法治政府、责任政府和廉洁政府。

　　各位代表，形势催人奋进，任务十分艰巨。让我们在市委坚强有力的领导下，同心同德，振奋精神，艰苦创业，奋力拼搏，不断开创各项工作新局面，为建设富民强市、和谐文明新揭阳，十年赶上全省发展平均水平而努力奋斗！
